# Supplementary material for: Structural basis of ribosomal 30S subunit degradation by RNase R
Source: Nature. 2024 Feb 7;626(8001):1133–40. doi: 10.1038/s41586-024-07027-6 (PMC10901742; doi:10.1038/s41586-024-07027-6)
Supplement: Supplementary file 1 — This file contains Supplementary Figs. 1–3 and Supplementary Tables 1–2. [file 41586_2024_7027_MOESM1_ESM.pdf]

nature portfolio

https://doi.org/10.1038/s41586-024-07027-6

Supplementary information

Structural basis of ribosomal 30S subunit degradation by RNase R

In the format provided by the authors and unedited

Supplementary Information for

Structural basis of ribosomal 30S subunit degradation by RNase R

Lyudmila Dimitrova-Paternoga[1], Sergo Kasvandik[2], Bertrand Beckert[3], Sander Granneman[4], Tanel Tenson[2], Daniel N. Wilson[1,*], Helge Paternoga[1,*]

[1] Institute for Biochemistry and Molecular Biology, University of Hamburg, Martin-Luther-King-Pl. 6, 20146 Hamburg, Germany

[2] University of Tartu, Institute of Technology, 50411 Tartu, Estonia

[3] Dubochet Center for Imaging (DCI) at EPFL, EPFL SB IPHYS DCI, Lausanne, Switzerland

[4] Centre for Engineering Biology (SynthSys), University of Edinburgh, Edinburgh, Scotland

*Correspondence to:

Helge Paternoga (Helge.Paternoga@uni-hamburg.de)

Daniel Wilson (Daniel.Wilson@uni-hamburg.de)

Table of Contents

Supplementary Fig. 1. Source data

Supplementary Fig. 2. Purification of the RNase R-30S complex

Supplementary Fig. 3. Establishment of an RNase R *in vitro* degradation assay

Supplementary Table 1. Northern blot probe sequences

Supplementary Table 2. Primers used to create endogenously-tagged RNase R-FLAG

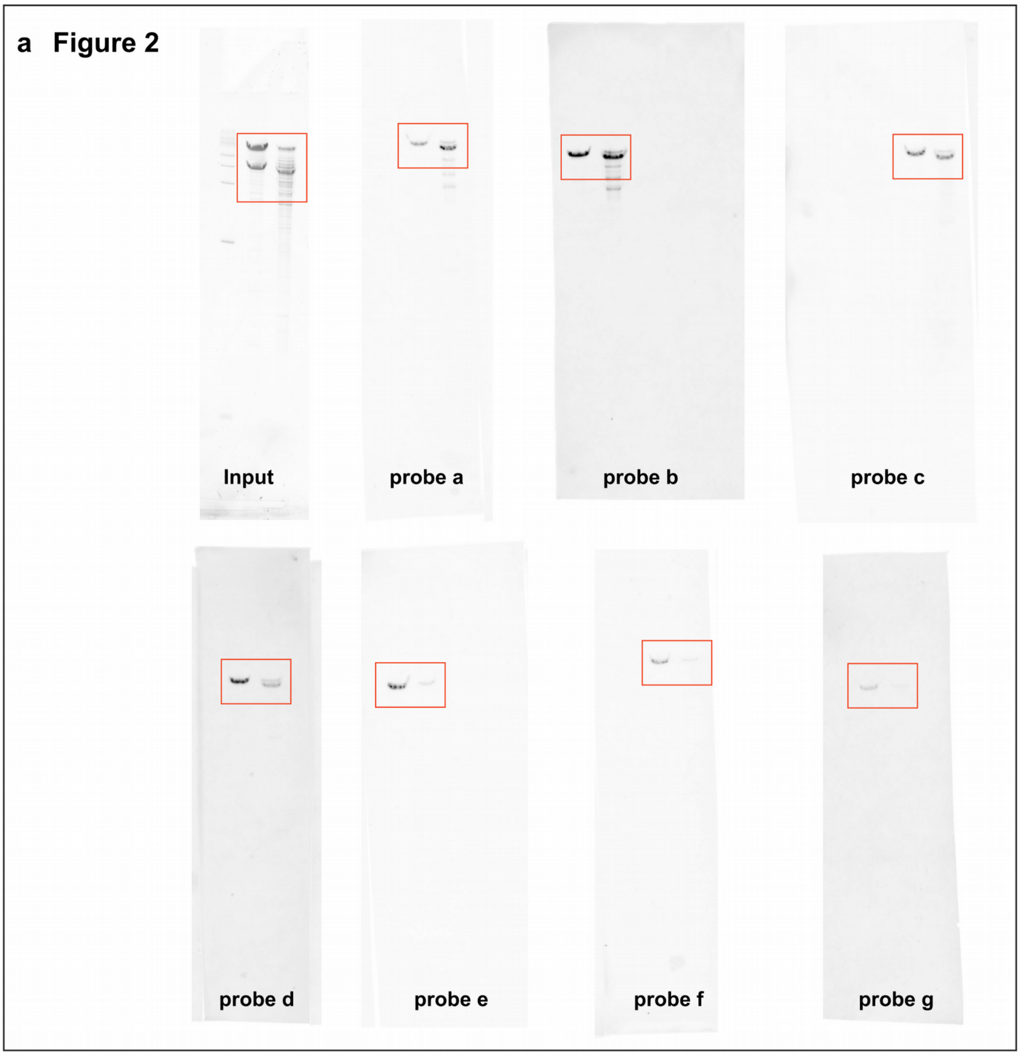
a Figure 2
Input
probe a
probe b
probe c
probe d
probe e
probe f
probe g

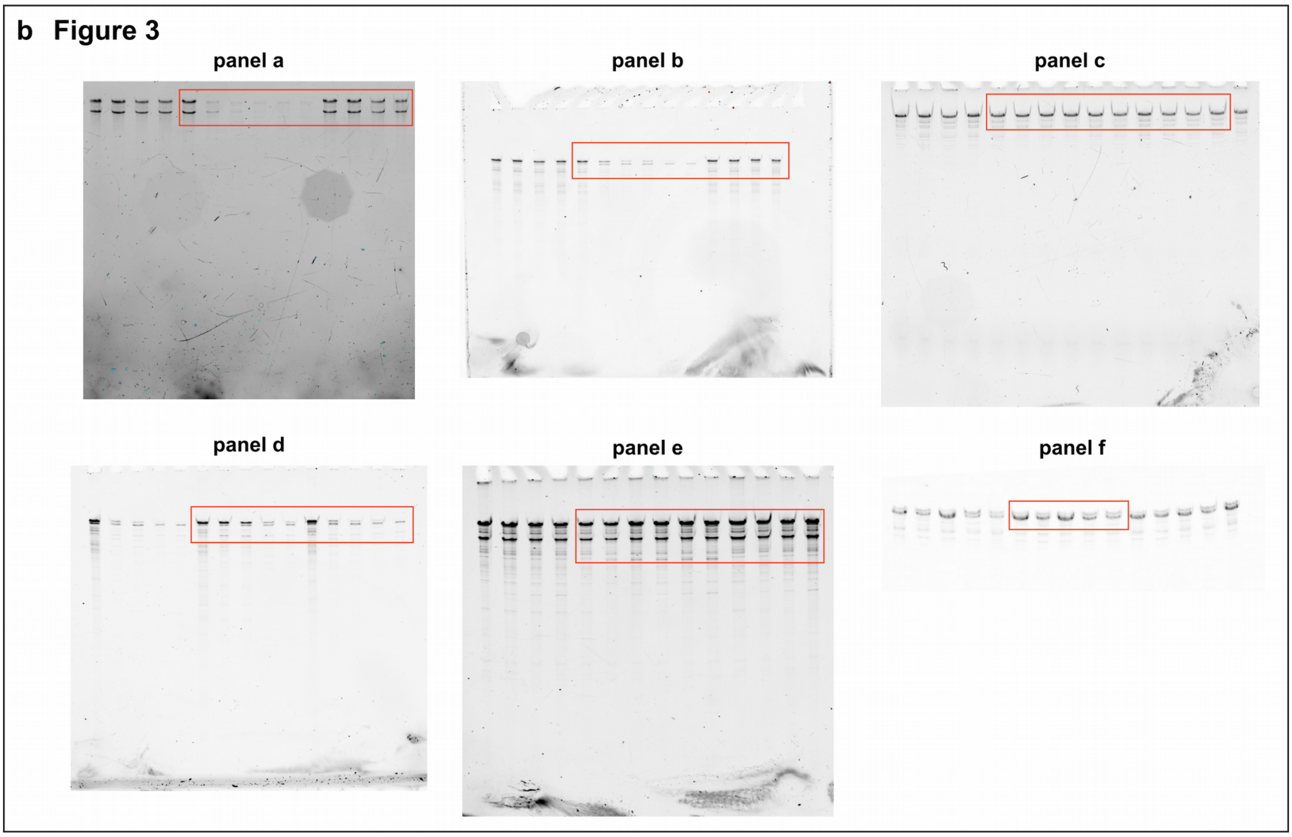
b Figure 3
panel a
panel b
panel c
panel d
panel e
panel f

c Extended Figure 7

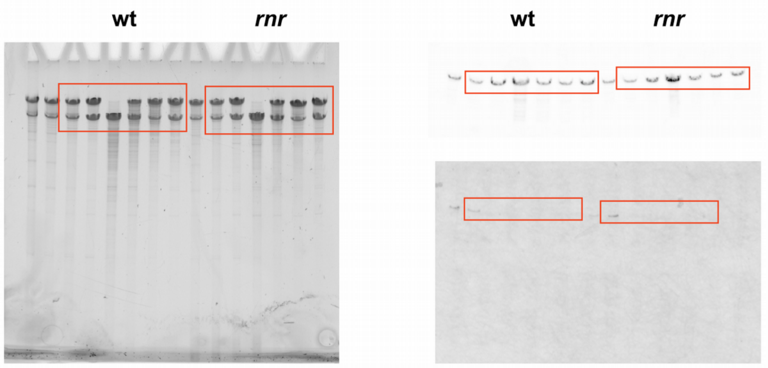

d Figure S2

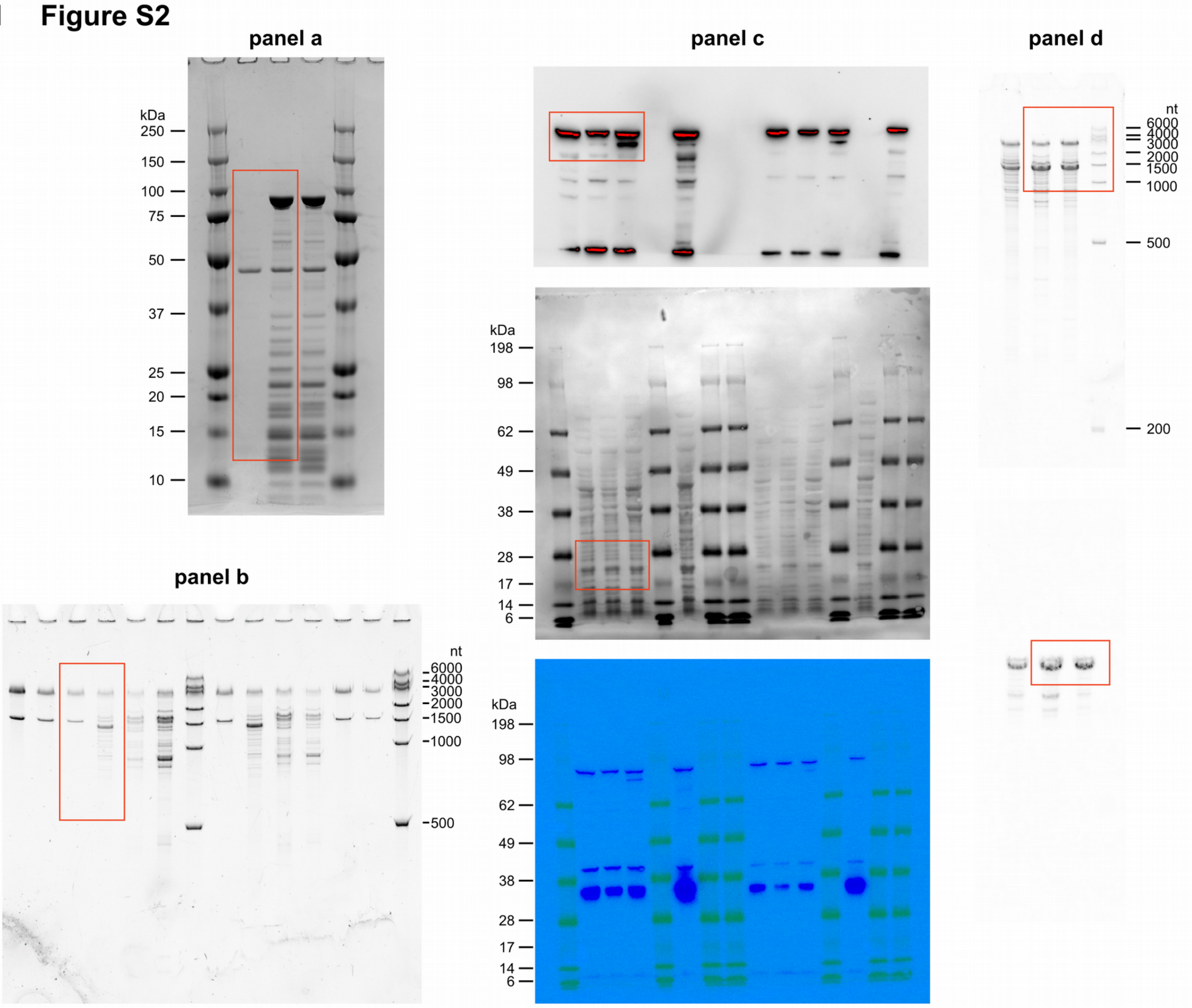

e Figure S3

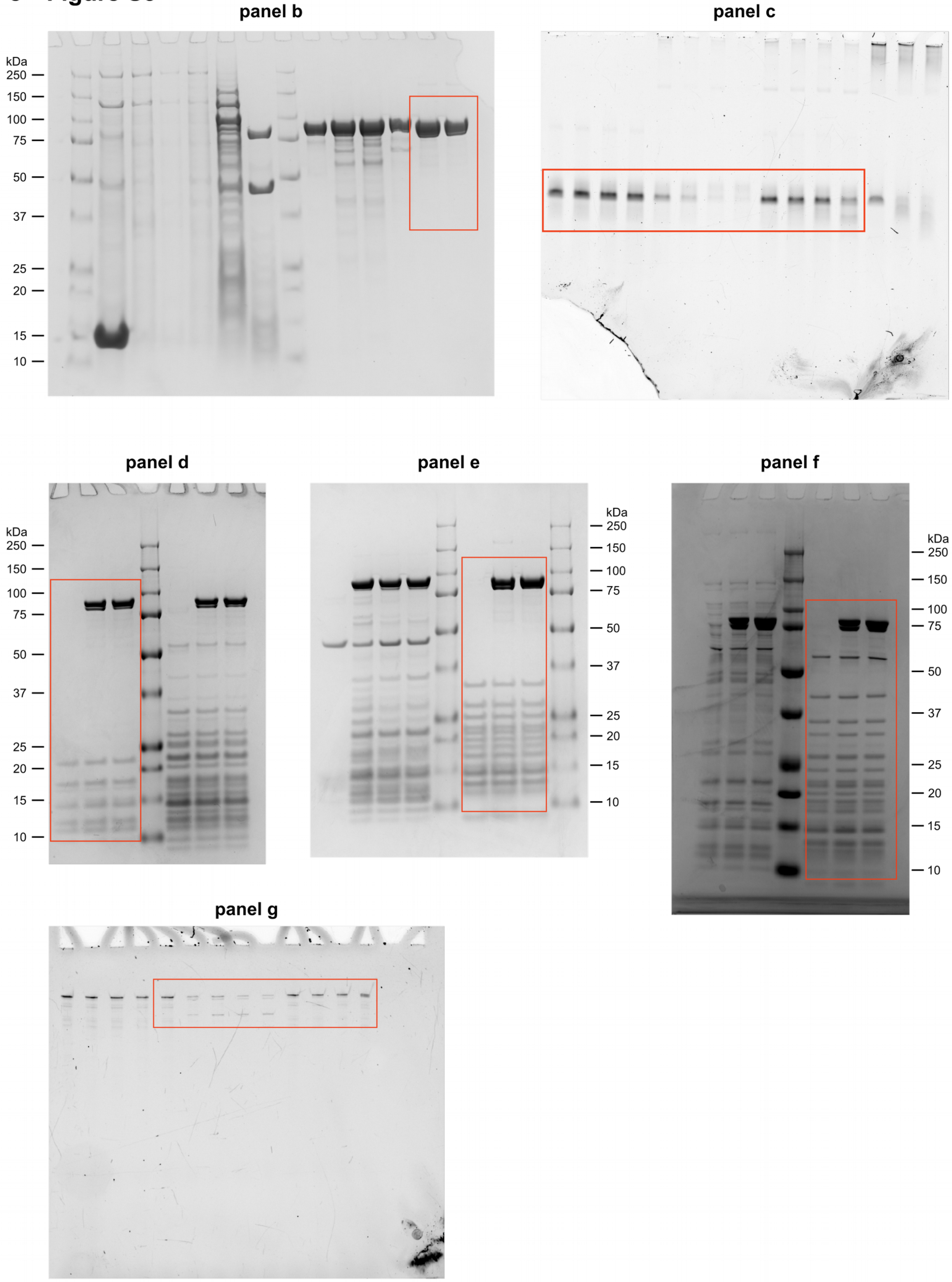

Supplementary Fig. 1. Source data. (a), Uncropped Northern blots used for Fig. 2. **(b)**, Uncropped gels used for Fig. 3. **(c)**, Uncropped gel and Northern blots used for Extended Figure 7. **(d)**, Uncropped gels, Northern and Western blots used for Supplementary Fig. 2. For the Western blot shown in panel c (top), saturated pixels are shown in red (the signal of interest is not saturated) **(e)**, Uncropped gels used for Supplementary Fig. 3.

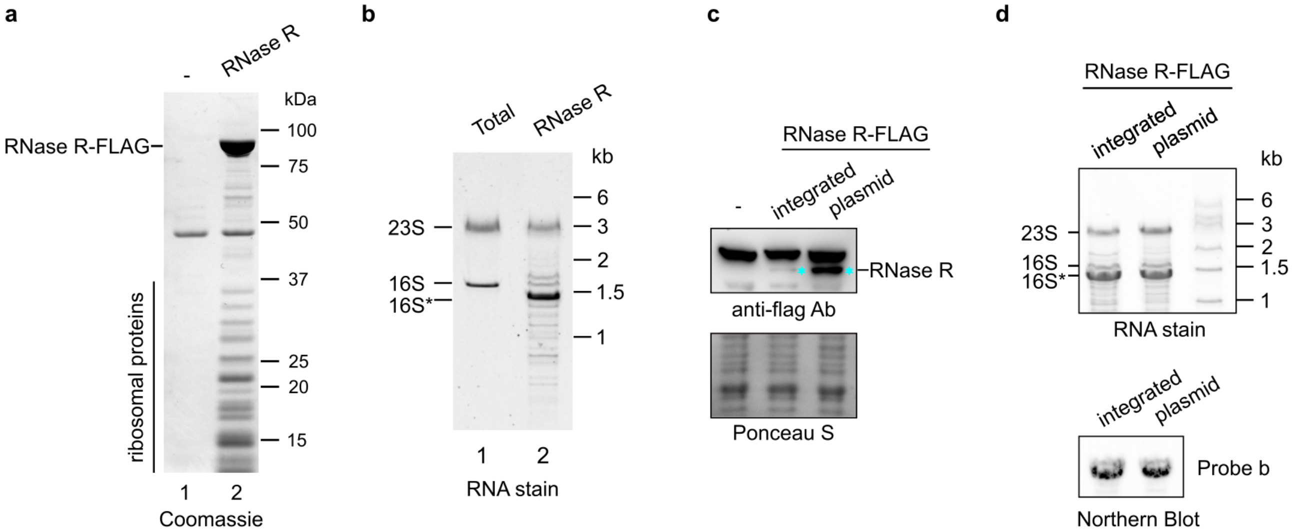

Supplementary Fig. 2. Purification of the RNase R-30S complex. (**a**) SDS-PAGE analysis of immuno-precipitated C-terminally FLAG-tagged RNase R (plasmid-based expression) (lane 2) as compared to a pull-down from vector-only control strain (lane 1) (n=5). (**b**) RNA analysis of immuno-precipitated C-terminally FLAG-tagged RNase R (plasmid-based expression) (n=5). Total RNA was loaded as a control. (**c**) Western blot analysis of wildtype strain (-), endogenously tagged RNase R-FLAG and a plasmid-borne RNase R-FLAG (expressed in *Δrnr* cells). The FLAG-tagged species were detected by HRP-coupled anti-FLAG Antibody (upper panel). The inputs were stained by Ponceau S as loading control (lower panel). The bands corresponding to RNase R-FLAG are indicated with an asterisk (two technical replicates were performed). (**d**) TBE-Urea polyacrylamide gel electrophoresis (PAGE) analysis of RNA extracted after immunoprecipitation of C-terminally FLAG-tagged RNase R, both from endogenous and plasmid-based expression. Equal amounts of RNA were loaded. Lower panel: Northern Blot analysis of the same RNA samples with probe b highlights the same truncation pattern of 16S rRNA (two technical replicates were performed). For gel source data, see Supplementary Fig. 1.

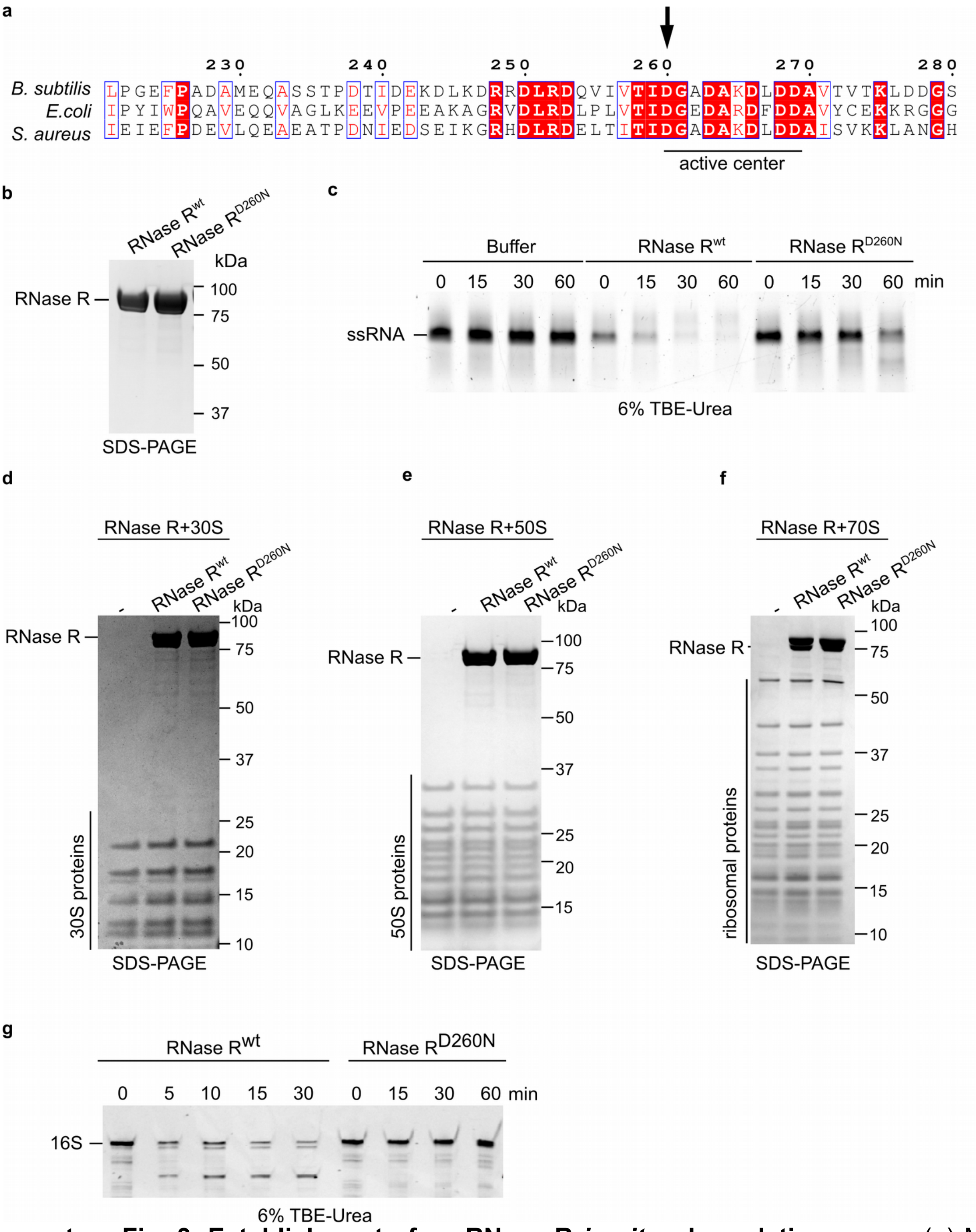

Supplementary Fig. 3. Establishment of an RNase R *in vitro* degradation assay. (**a**) Multiple sequence alignment of RNase R from *B. subtilis*, *E. coli* and *S. aureus.* The conserved active center of the enzyme is underlined. In *B. subtilis*, D260, D267 and D268 are predicted by homology to coordinate a Mg^{2+} ion with two waters (Chu *et al.* "Structural Insights into RNA Unwinding and Degradation by RNase R." Nucleic Acids Research 45, no. 20 (November 16, 2017): 12015–24. https://doi.org/10.1093/nar/gkx880). (**b**) SDS-PAGE analysis of recombinantly expressed and purified wildtype RNase R (Rnr^{wt}) and catalytically inactive RNase R-D260N mutant (Rnr^{D260N}) (n=3). (**c**) *In vitro* degradation assay using linear RNA as a substrate. RNase R was mixed with the substrate and incubated at 37°C for 0-60 mins, after which the RNA was analyzed on 6 % denaturing TBE-Urea gels (n=2). (**d-f**) Inputs for the assays in Figure 3 c, d, f, respectively (n=2). (**g**) *In vitro* degradation assay of isolated 30S ribosomal subunits, catalyzed by recombinantly purified RNase R wt and catalytic mutant in a buffer containing 15 mM $MgCl_2$. RNase R was mixed with the substrate and incubated at 37°C for the above indicated times after which the RNA was extracted and analyzed on 6 % denaturing TBE-Urea gel (two technical replicates were performed). For gel source data, see Supplementary Fig. 1.

Supplementary Table 1 Northern blot probe sequences

Probe	Boundaries	Sequence
a	22 - 41	CCAGCGTTCGTCCTGAGCCA
b	652 - 670	CTGCACTCAAGTTCCCCAG
c	1372 - 1391	GAACGTATTCACCGCGGCAT
d	1392 - 1409	GGTGTGTACAAGGCCCGG
e	1412 - 1432	CAAACTCTCGTGGTGTGACGG
f	1422 - 1441	CGGGTGTTACAAACTCTCGT
g	1527 - 1549	GGAGGTGATCCAGCCGCACCTTC
3‘ pre<br><br>(1:1 mixture)	+1 - +30<br><br>+43 - +57	CCAAGGTCTTATATTCCGTAAAATATCCTT<br><br>CTAAACAAGACAGGGAACGTTC

Supplementary Table 2 Primers used to create endogenously-tagged RNase R-FLAG

iPCR_GS5-FLAG-CAT_fw	gattataaagatgatgatgataaaTAAGCCTCCTAAATTCACTTTAGATAAAAATTTAGGAG
iPCR_GS5-FLAG-CAT_rv	gcttcctgagccgcttcctgagccGgatccCTTGTCTGCTTTCTTCATTAGAATCAATCC
over1_fw	CAACGGAAAGAATGACATATTCAGATGTG
over1_rv	gcttcctgagccggatccTTTCTTCTTTTTCCGTTTCTGTTTCG
over2_fw	ggatccggctcaggaagc
over2_rv	TTATAAAAGCCAGTCATTAGGCCTATC
over3_fw	GATAGGCCTAATGACTGGCTTTTATAAcagctcaaccagcaaataggg
over3_rv	GCTGGGATTGAAATGGAAGTACG
COL_fw	GACAATTAACAGTCAGGGACAGG
COL_rv	ATTCAGCTTCATTATTCATCCCTCC
